# Clinical predictive modeling for post-ERCP cholangitis in biliary stricture patients

**DOI:** 10.3389/fmed.2026.1738706

**Published:** 2026-04-22

**Authors:** Bensong Duan, Weixin Ye, Yuping Shi, Suhong Yi, Dadong Wan, Yang Jiang, Liqian Xuan, Jiangfeng Hu

**Affiliations:** 1 Endoscopy Center, Department of Gastroenterology, Shanghai East Hospital, Tongji University School of Medicine, Shanghai, China; 2 Department of Gastroenterology, Xining Second People’s Hospital, Xining City, Qinghai, China; 3 Department of Nephrology, Shanghai Tongren Hospital, Shanghai Jiao Tong University School of Medicine, Shanghai, China; 4 Department of Gastroenterology, Xinyu People’s Hospital, Xinyu, Jiangxi, China; 5 Department of Gastroenterology, Fuyang Women & Children’s Hospital, Fuyang, Anhui, China; 6 Department of Neurosurgery, Shanghai Tenth People’s Hospital, Tongji University School of Medicine, Shanghai, China; 7 Department of Gastroenterology, Shuguang Hospital Affiliated to Shanghai University of Traditional Chinese Medicine, Shanghai, China; 8 Department of Gastroenterology, Shanghai General Hospital, Shanghai Jiao Tong University School of Medicine, Shanghai, China

**Keywords:** biliary stricture, ERCP, nomogram, post-ERCP cholangitis, predictive model

## Abstract

**Aim:**

The objective of this study was to predict the occurrence of post-endoscopic retrograde cholangiopancreatography (ERCP) cholangitis (PEC) in patients with biliary stricture.

**Method:**

We collected clinical data and procedural records from 1,606 patients who underwent ERCP for biliary stricture over the last 10 years. Of these, 1,281 patients were randomly allocated to the training or validation group and 325 patients enrolled in other hospitals as an external validation set. LASSO logistic regression analysis was used to identify independent risk factors and establish a predictive model for PEC. The performance of the nomogram was evaluated using receiver operating characteristic (ROC) curves and calibration curves. At the same time, decision curve analysis (DCA) is used to determine the net benefit threshold of prediction.

**Results:**

The findings indicated that age, albumin levels, etiology of biliary stricture, stenosis site, diabetes, digestive tract reconstruction, obstruction length and the use of cholangioscopy may be factors that influence postoperative biliary tract infection in patients with biliary stenosis. The nomogram exhibited a higher net benefit in decision curve analysis. The calibration curve shows a strong agreement between expected and observed results.

**Conclusion:**

The proposed nomogram exhibits robust predictive performance. This tool can assist healthcare professionals in minimizing the risk of PEC in patients with biliary stricture undergoing ERCP.

## Introduction

Patients with biliary stricture often require endoscopic retrograde cholangiopancreatography (ERCP) procedures for diagnosis and treatment (1–4). This group of patients is at an increased risk of developing post-ERCP complications such as cholangitis, specifically an inflammatory condition of the bile ducts (5). Cholangitis can be a serious and potentially life-threatening infection that requires prompt recognition and appropriate management.

Several factors, such as age over 60, history of ERCP, and primary sclerosing cholangitis (PSC), have been identified as potential predictors of post-ERCP cholangitis (PEC) in unselected patients (6, 7). However, evidence specific to biliary stricture populations remains limited.

To better understand and predict the occurrence of PEC in patients with biliary strictures, our study aims to conduct a clinical prediction study focusing on this specific complication. We will analyze a range of potential predictive factors including demographic characteristics such as Sex and Age, laboratory parameters like serum albumin levels, comorbidities such as diabetes, anatomical features including biliary stricture site and biliary obstruction length, and procedural variables such as duration of the ERCP procedure and the use of advanced imaging modalities like Spyglass cholangioscopy.

Logistic regression analysis will be conducted to identify independent predictors of post-ERCP cholangitis in this patient population. Additionally, a nomogram will be developed to provide a graphical representation of the predictive model, allowing clinicians to easily calculate an individual patient’s risk of developing cholangitis following ERCP.

By elucidating the factors that influence the occurrence of post-ERCP cholangitis in patients with biliary strictures, our study aims to provide valuable insights for clinical decision-making and risk stratification in this high-risk group of patients.

## Materials and methods

This study was a multicenter retrospective study that enrolled patients undergoing ERCP for biliary strictures. Inclusion criteria: Patients diagnosed with biliary stricture who had underwent ERCP. Exclusion criteria: 1. Patients with fever or elevated white blood cells before ERCP, 2. Patients who developed post-ERCP complications other than cholangitis, 3. Patients with incomplete medical records. A total of 2,318 patients were initially considered; among them, 132 had incomplete data, 307 exhibited preoperative leukocytosis or fever, and 273 developed postoperative complications other than cholangitis. Ultimately, 1,606 patients were included in this study. A participant flow diagram was shown in Supplementary Figure 1.

The following demographic and clinical data were obtained: Sex, Age (years), Wbc (white blood cell, *10^9^/L), Hb (Hemoglobin, g/L), Alt (alanine aminotransferase, U/L), Tbil (total bilirubin, μmol/L), GGT (gamma-glutamyl transferase, U/L), Alb (albumin, g/L), CEA (carcinoembryonic Antigen), CA19-9 (carbohydrate antigen 19-9), Etiologie, Digestive tract reconstruction, Gallstone, Stenosis site, Diabetes, Obstruction length, ERCP operation time (hour, h), EST (Endoscopic Sphincterotomy), EPBD (Endoscopic Papillary Balloon Dilatation), ENBD (Endoscopic Nasobiliary Drainage), ERBD (Endoscopic Retrograde Biliary Drainage), Cytology Brush, EMBE (Endoscopic Metal Biliary Endoprosthesis), IDUS (Intraductal Ultrasonography), operators, and cholangioscopy. The data of the outcome, cholangitis, were also collected.

The dataset collected was randomly divided into training and validation groups. The data of the training set and the internal validation set came from Shanghai General Hospital. To validate the model, data from the external validation set were collected from five hospitals in different regions of China. Non-normally distributed data were expressed as median (interquartile ranges). Univariate analysis used chi-square test or Fisher’s exact test for categorical variables, and Student’s *t*-test or rank-sum test for continuous variables. In the training group, LASSO logistic regression analysis was used for multivariate analysis to identify independent risk factors and create a prediction nomogram for cholangitis. The performance of the nomogram was assessed using the ROC curve and calibration curve, with the AUC ranging from 0.5 to 1. Decision curve analysis was also performed to determine the net benefit threshold of prediction. Results with a *p*-value <0.05 were considered statistically significant. All statistical analyses were conducted using R software (version 4.2.2).

## Results

### Patient characteristics

A total of 1,606 patients were included in this study and were divided into three cohorts: a training cohort (*N* = 769), an internal test cohort (*N* = 512), and an external test cohort (*N* = 325). The baseline demographic, clinical, and procedural characteristics of these cohorts are summarized in Table 1. The distribution of sex was comparable across the three groups, with a *p*-value of 0.092. In the training cohort, 45.5% were female and 54.5% were male; the internal test cohort comprised 51.4% females and 48.6% males; and the external test cohort included 50.2% females and 49.8% males. The age distribution, categorized as <60 years or ≥60 years, was also similar among the cohorts (*p* = 0.778). The proportion of patients aged <60 years was 55.5% in the training cohort, 56.1% in the internal test cohort, and 57.8% in the external test cohort, with the corresponding proportions for those aged ≥60 years being 44.5, 43.9, and 42.2%, respectively.

**Table 1:** Patient demographics and baseline characteristics.

Characteristic	Cohort (training cohort), *N* = 769			Cohort (internal test cohort), *N* = 512			Cohort (external test cohort), *N* = 325		
	Cholangitis (No), *N* = 618^a^	Cholangitis (Yes), *N* = 151^a^	*p*-value^b^	Cholangitis (No), *N* = 427^a^	Cholangitis (Yes), *N* = 85^a^	*p*-value^c^	Cholangitis (No), *N* = 271^a^	Cholangitis (Yes), *N* = 54^a^	*p*-value^c^
Sex			0.131			0.268			0.535
Female	273 (44.2%)	77 (51.0%)		224 (52.5%)	39 (45.9%)		138 (50.9%)	25 (46.3%)	
Male	345 (55.8%)	74 (49.0%)		203 (47.5%)	46 (54.1%)		133 (49.1%)	29 (53.7%)	
Age (years)			0.011			0.001			0.114
<60	357 (57.8%)	70 (46.4%)		253 (59.3%)	34 (40.0%)		162 (59.8%)	26 (48.1%)	
≥60	261 (42.2%)	81 (53.6%)		174 (40.7%)	51 (60.0%)		109 (40.2%)	28 (51.9%)	
Wbc (×10^9^/L)	5.75 ± 1.78	5.87 ± 1.65	0.423	5.71 ± 1.76	6.38 ± 1.56	<0.001	5.09 ± 1.66	5.67 ± 1.87	0.036
Hb (g/L)			0.468			0.781			0.601
<110	199 (32.2%)	44 (29.1%)		127 (29.7%)	24 (28.2%)		140 (51.7%)	30 (55.6%)	
≥110	419 (67.8%)	107 (70.9%)		300 (70.3%)	61 (71.8%)		131 (48.3%)	24 (44.4%)	
Alt (U/L)	160 ± 123	155 ± 121	0.667	169 ± 123	185 ± 121	0.272	98 ± 114	115 ± 127	0.364
Tbil (μmol/L)	179 ± 107	181 ± 106	0.860	191 ± 106	169 ± 109	0.096	119 ± 121	140 ± 135	0.285
GGT (U/L)	456 ± 277	466 ± 300	0.713	456 ± 296	473 ± 283	0.617	311 ± 304	452 ± 522	0.059
CEA	5 (3, 107)	12 (4, 90)	0.041	6 (3, 94)	20 (3, 146)	0.185	4 (3, 23)	8 (3, 55)	0.050
Alb (g/L)			<0.001			0.019			<0.001
<35	331 (53.6%)	112 (74.2%)		227 (53.2%)	57 (67.1%)		89 (32.8%)	35 (64.8%)	
≥35	287 (46.4%)	39 (25.8%)		200 (46.8%)	28 (32.9%)		182 (67.2%)	19 (35.2%)	
CA19-9	177 (17, 536)	178 (20, 615)	0.895	126 (18, 520)	326 (19, 579)	0.233	55 (16, 299)	64 (19, 691)	0.140
Etiologie									
Ampullarycarcinoma	42 (6.8%)	5 (3.3%)		38 (8.9%)	3 (3.5%)		33 (12.2%)	2 (3.7%)	
Autoimmune	15 (2.4%)	7 (4.6%)		8 (1.9%)	3 (3.5%)		17 (6.3%)	0 (0.0%)	
Benign strictures	201 (32.5%)	22 (14.6%)		151 (35.4%)	20 (23.5%)		39 (14.4%)	2 (3.7%)	
d-CCA	54 (8.7%)	11 (7.3%)		31 (7.3%)	2 (2.4%)		34 (12.5%)	10 (18.5%)	
GBC	29 (4.7%)	10 (6.6%)		14 (3.3%)	4 (4.7%)		9 (3.3%)	3 (5.6%)	
h-CCA	84 (13.6%)	38 (25.2%)		57 (13.3%)	23 (27.1%)		38 (14.0%)	15 (27.8%)	
HCC	29 (4.7%)	14 (9.3%)		22 (5.2%)	8 (9.4%)		9 (3.3%)	6 (11.1%)	
Iatrogenic	17 (2.8%)	5 (3.3%)		19 (4.4%)	3 (3.5%)		18 (6.6%)	1 (1.9%)	
Metastatic tumors	32 (5.2%)	12 (7.9%)		11 (2.6%)	6 (7.1%)		20 (7.4%)	7 (13.0%)	
Pancreatic cancer	115 (18.6%)	27 (17.9%)		76 (17.8%)	13 (15.3%)		54 (19.9%)	8 (14.8%)	
Digestive tract reconstruction			<0.001			<0.001			0.287
No	603 (97.6%)	135 (89.4%)		419 (98.1%)	74 (87.1%)		260 (95.9%)	50 (92.6%)	
Yes	15 (2.4%)	16 (10.6%)		8 (1.9%)	11 (12.9%)		11 (4.1%)	4 (7.4%)	
Gallstone			0.264			0.892			0.314
No	400 (64.7%)	105 (69.5%)		273 (63.9%)	55 (64.7%)		232 (85.6%)	49 (90.7%)	
Yes	218 (35.3%)	46 (30.5%)		154 (36.1%)	30 (35.3%)		39 (14.4%)	5 (9.3%)	
Stenosis site			<0.001			<0.001			<0.001
Extrahepatic	425 (68.8%)	73 (48.3%)		286 (67.0%)	39 (45.9%)		198 (73.1%)	23 (42.6%)	
Hilar/intrahepatic	193 (31.2%)	78 (51.7%)		141 (33.0%)	46 (54.1%)		73 (26.9%)	31 (57.4%)	
Diabetes			<0.001			<0.001			0.050
No	521 (84.3%)	105 (69.5%)		362 (84.8%)	59 (69.4%)		214 (79.0%)	36 (66.7%)	
Yes	97 (15.7%)	46 (30.5%)		65 (15.2%)	26 (30.6%)		57 (21.0%)	18 (33.3%)	
Obstruction length (cm)			<0.001			0.015			<0.001
˃2.5	252 (40.8%)	85 (56.3%)		170 (39.8%)	46 (54.1%)		56 (20.7%)	30 (55.6%)	
≤2.5	366 (59.2%)	66 (43.7%)		257 (60.2%)	39 (45.9%)		215 (79.3%)	24 (44.4%)	
ERCP operation time (h)			0.185			<0.001			<0.001
<1	348 (56.3%)	76 (50.3%)		249 (58.3%)	33 (38.8%)		201 (74.2%)	22 (40.7%)	
≥1	270 (43.7%)	75 (49.7%)		178 (41.7%)	52 (61.2%)		70 (25.8%)	32 (59.3%)	
EST			0.347			0.057			0.775
No	175 (28.3%)	37 (24.5%)		129 (30.2%)	17 (20.0%)		41 (15.1%)	9 (16.7%)	
Yes	443 (71.7%)	114 (75.5%)		298 (69.8%)	68 (80.0%)		230 (84.9%)	45 (83.3%)	
ENBD			0.154			0.501			0.283
No	235 (38.0%)	48 (31.8%)		184 (43.1%)	40 (47.1%)		127 (46.9%)	21 (38.9%)	
Yes	383 (62.0%)	103 (68.2%)		243 (56.9%)	45 (52.9%)		144 (53.1%)	33 (61.1%)	
EPBD			0.168			0.460			0.462
No	162 (26.2%)	48 (31.8%)		138 (32.3%)	24 (28.2%)		35 (12.9%)	9 (16.7%)	
Yes	456 (73.8%)	103 (68.2%)		289 (67.7%)	61 (71.8%)		236 (87.1%)	45 (83.3%)	
ERBD			0.337			0.321			0.785
No	321 (51.9%)	85 (56.3%)		206 (48.2%)	36 (42.4%)		135 (49.8%)	28 (51.9%)	
Yes	297 (48.1%)	66 (43.7%)		221 (51.8%)	49 (57.6%)		136 (50.2%)	26 (48.1%)	
Cytology brush			0.605			0.781			<0.001
No	317 (51.3%)	81 (53.6%)		213 (49.9%)	41 (48.2%)		173 (63.8%)	20 (37.0%)	
Yes	301 (48.7%)	70 (46.4%)		214 (50.1%)	44 (51.8%)		98 (36.2%)	34 (63.0%)	
EMBE			0.039			0.452			0.580
No	348 (56.3%)	99 (65.6%)		235 (55.0%)	43 (50.6%)		217 (80.1%)	45 (83.3%)	
Yes	270 (43.7%)	52 (34.4%)		192 (45.0%)	42 (49.4%)		54 (19.9%)	9 (16.7%)	
IDUS			0.890			0.580			0.980
No	319 (51.6%)	77 (51.0%)		202 (47.3%)	43 (50.6%)		140 (51.7%)	28 (51.9%)	
Yes	299 (48.4%)	74 (49.0%)		225 (52.7%)	42 (49.4%)		131 (48.3%)	26 (48.1%)	
Cholangioscopy			<0.001			<0.001			<0.001
No	457 (73.9%)	77 (51.0%)		327 (76.6%)	36 (42.4%)		211 (77.9%)	21 (38.9%)	
Yes	161 (26.1%)	74 (49.0%)		100 (23.4%)	49 (57.6%)		60 (22.1%)	33 (61.1%)	

a *n* (%); Mean ± SD; Median (Q1, Q3).

b Pearson’s Chi-squared test; Welch Two Sample *t*-test; Wilcoxon rank sum test.

c Pearson’s Chi-squared test; Welch Two Sample *t*-test; Wilcoxon rank sum test; Fisher’s exact test.

Several laboratory parameters showed significant differences across the cohorts. The median white blood cell count (WBC) was 6.00 (Q1-Q3: 4.33–7.00) ×10^9^/L in the training cohort, 6.00 (4.30–7.00) in the internal test cohort, and 5.00 (4.00–6.60) in the external test cohort (*p* < 0.001). Similarly, median alanine aminotransferase (ALT) levels were 132 U/L (46–263) in the training cohort, 159 U/L (53–278) in the internal test cohort, and notably lower at 55 U/L (26–128) in the external test cohort (*p* < 0.001). Hemoglobin (Hb) levels, dichotomized at 110 g/L, demonstrated a significant disparity (*p* < 0.001). A lower proportion of patients in the external test cohort (52.3%) had Hb < 110 g/L compared to the training (31.6%) and internal test (29.5%) cohorts. Conversely, the proportion with Hb ≥ 110 g/L was 47.7% in the external test cohort versus 68.4 and 70.5% in the training and internal test cohorts, respectively. Total bilirubin (Tbil) levels also differed significantly (*p* < 0.001), with median values of 171 μmol/L (80–264) in the training cohort, 171 μmol/L (90–278) in the internal test cohort, and 74 μmol/L (26–187) in the external test cohort. Albumin (Alb) levels, categorized as <35 g/L or ≥35 g/L, showed a significant distribution (*p* < 0.001). The proportion of patients with Alb <35 g/L was 57.6% in the training cohort, 55.5% in the internal test cohort, and 38.2% in the external test cohort. Median carcinoembryonic antigen (CEA) levels were 6 (3–103) in the training cohort, 6 (3–109) in the internal test cohort, and 4 (3–31) in the external test cohort (*p* < 0.001). Median carbohydrate antigen 19–9 (CA19-9) levels were 178 (17–547) U/mL, 141 (18–539) U/mL, and 57 (16–347) U/mL in the training, internal test, and external test cohorts, respectively (*p* = 0.038). Median gamma-glutamyl transferase (GGT) levels were 430 U/L (221–695) in the training cohort, 451 U/L (233–674) in the internal test cohort, and 257 U/L (76–473) in the external test cohort (*p* < 0.001).

The etiological composition of biliary obstruction varied significantly among the cohorts (*p* < 0.001). In the training cohort, the most common etiologies were benign strictures (29.0%), pancreatic cancer (18.5%), and hilar cholangiocarcinoma (h-CCA, 15.9%). The internal test cohort had a similar profile: benign strictures (33.4%), pancreatic cancer (17.4%), and h-CCA (15.6%). The external test cohort, however, showed a different pattern, with a lower prevalence of benign strictures (12.6%) and higher proportions of ampullary carcinoma (10.8%) and distal cholangiocarcinoma (d-CCA, 13.5%). The prevalence of gallstone disease differed markedly (*p* < 0.001), with only 13.5% of patients in the external test cohort having gallstones compared to 34.3% in the training and 35.9% in the internal test cohorts. The site of stenosis, categorized as extrahepatic or hilar/intrahepatic, was not significantly different across groups (*p* = 0.401). The proportion of patients with an obstruction length >2.5 cm was significantly lower in the external test cohort (26.5%) compared to the training (43.8%) and internal test (42.2%) cohorts (*p* < 0.001). The distribution of ERCP operation time, categorized as <1 h or ≥1 h, also differed (*p* < 0.001), with a higher proportion of procedures lasting <1 h in the external test cohort (68.6%) versus the other two cohorts (both 55.1%).

Procedural characteristics revealed several inter-cohort variations. The rates of endoscopic sphincterotomy (EST) were 72.4, 71.5, and 84.6% in the training, internal test, and external test cohorts, respectively (*p* < 0.001). Endoscopic papillary balloon dilation (EPBD) was performed in 72.7, 68.4, and 86.5% of cases across the three cohorts (*p* < 0.001). The use of endoscopic nasobiliary drainage (ENBD) was 63.2% in the training cohort, 56.3% in the internal test cohort, and 54.5% in the external test cohort (*p* = 0.007). The rates of endoscopic retrograde biliary drainage (ERBD) placement were 47.2, 52.7, and 49.8%, showing no significant difference (*p* = 0.151). Cytology brushing was performed in 48.2, 50.4, and 40.6% of cases in the three cohorts (*p* = 0.018). Endoscopic metal biliary endoprosthesis (EMBE) placement was used significantly less frequently in the external test cohort (19.4%) compared to the training (41.9%) and internal test (45.7%) cohorts (*p* < 0.001). The utilization rates of intraductal ultrasonography (IDUS) and cholangioscopy were not significantly different across the cohorts, with *p*-values of 0.384 and 0.762, respectively. The prevalence of a history of digestive tract reconstruction and diabetes mellitus was also comparable among the groups, with *p*-values of 0.811 and 0.136, respectively.

### Outcome assessment and definitions

PEC was defined as the onset of a new fever (body temperature >38 °C) accompanied by an elevated white blood cell count, increased C-reactive protein levels, and worsening liver function tests, persisting for more than 24 h after ERCP (6, 7). Cases involving pulmonary infection, acute pancreatitis, and other unrelated infections were excluded. Cholangitis was classified as mild, moderate, or severe according to the Tokyo Guidelines (8). Mild cases were managed with antibiotics alone, while moderate cases required early biliary reintervention, such as endoscopic or percutaneous drainage. All severity grades were included in the study. Statistical analysis shows that the majority of the cases were mild, and antibiotic treatment was effective. However, we observed that some patients with biliary strictures required prolonged antibiotic treatment after ERCP, frequently guided by bile or blood culture results. These individuals showed a higher propensity for infections with resistant bacteria, such as *Enterococcus faecalis* and *Enterococcus faecium*, necessitating the use of broader-spectrum or more advanced antibiotics (9–11). The grading of inflammation severity in PEC patients, the duration of antibiotic use, and the results of bacterial culture were shown in Supplementary Tables S1–S3.

### Predictive model

**Figure 1:**
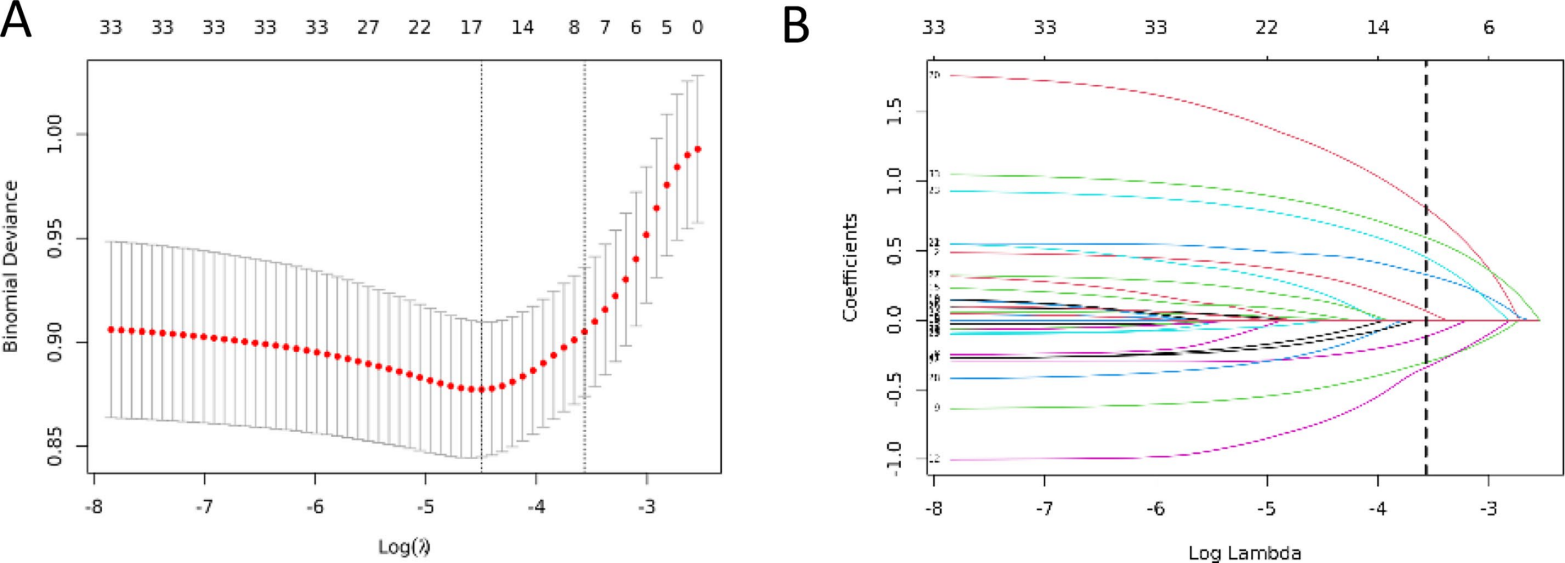
Identification of the risk factors of PEC by LASSO regression. **(A)** Ten-fold cross-validation was applied to select the most suitable feature using the LASSO regression model. **(B)** A coefficient profile plot was produced against the log(lambda) sequence.

**Figure 2:**
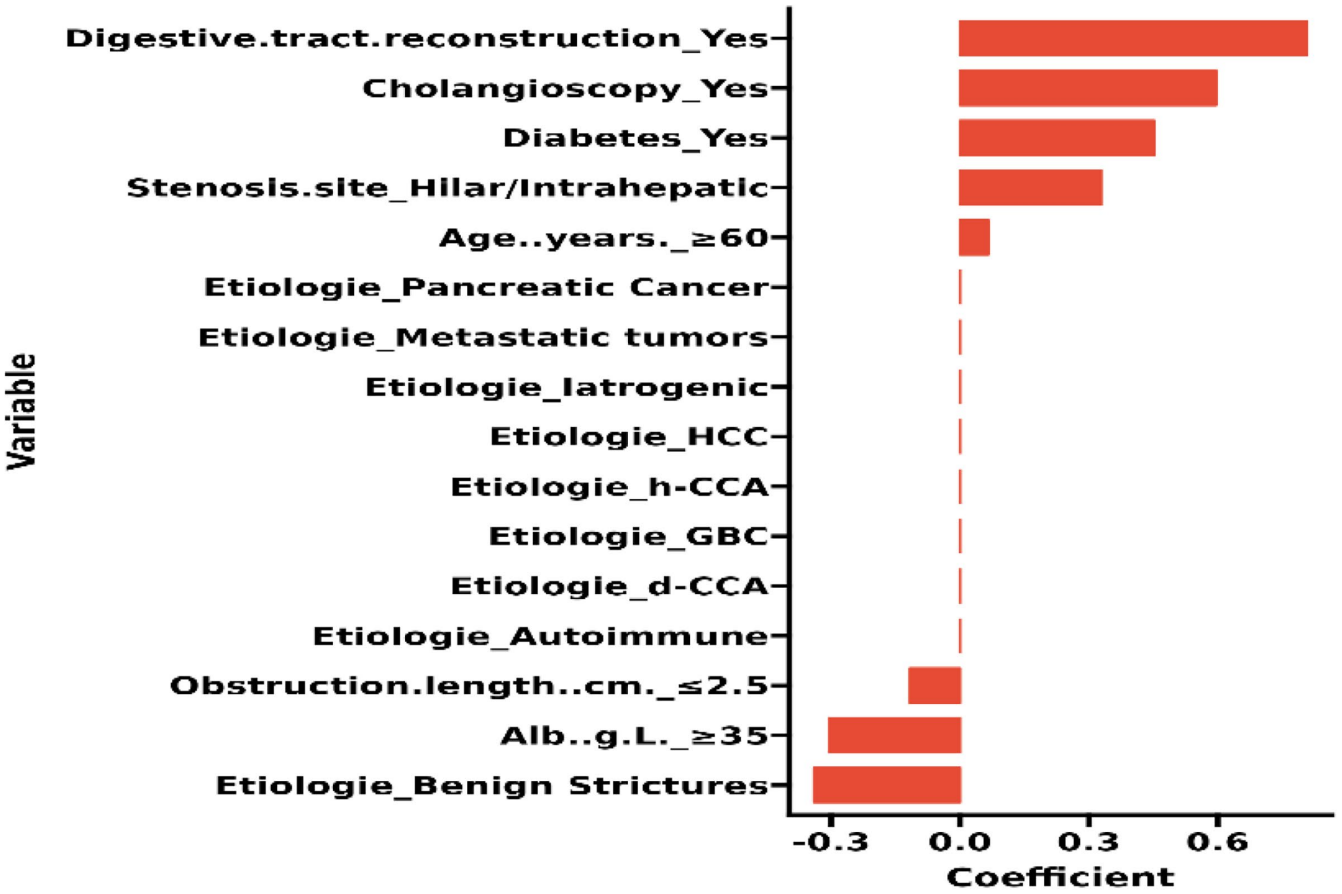
Histogram of the coefficients of the selected features.

**Figure 3:**
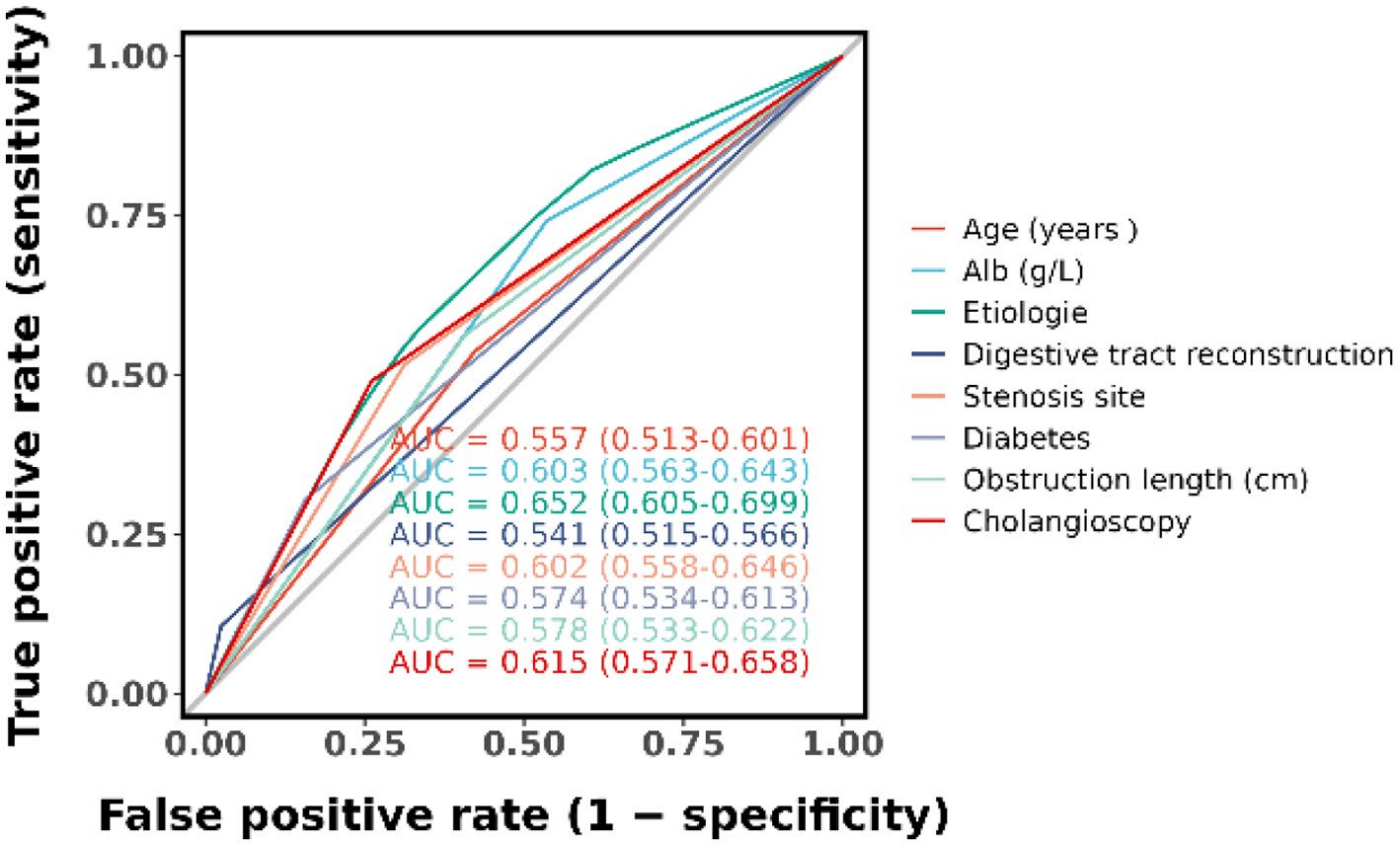
ROC curve analysis of candidate diagnostic indicators.

**Figure 4:**
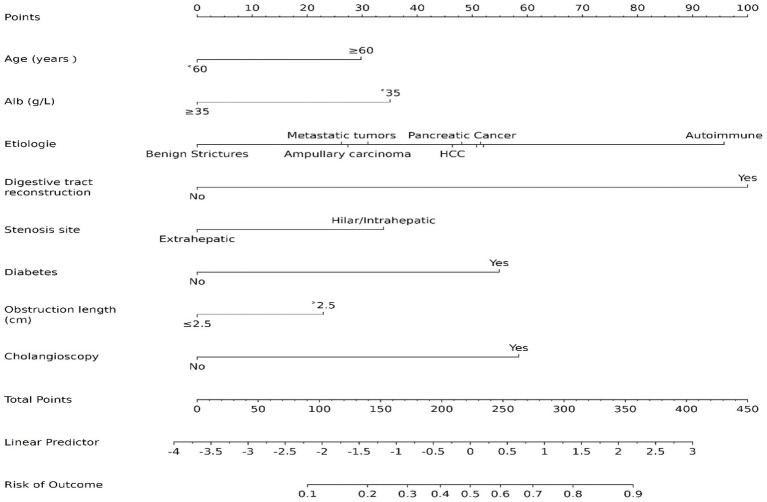
Nomogram prediction model.

The candidate predictors, Sex, Age, Wbc, Hb, Alt, Tbil, GGT, Alb, CEA, CA19-9, etiology, gallstone, digestive tract reconstruction, stenosis site, diabetes, obstruction length, ERCP operation time, EST, EPBD, ENBD, ERBD, Cytology Brush, EMBE, Spyglass, operators, and IDUS, were included in the original model, which was then reduced to 8 potential predictors using LASSO regression analysis performed in the training cohort. A cross-validated error plot of the LASSO regression model is shown in Figure 1. The most regularized and parsimonious model, with a cross-validated error within one standard error of the minimum, included 8 variables. The details of the selected features are shown in Figure 2. As shown in Figure 3, the ROC analysis of the abovementioned variables yielded AUC values greater than 0.5. Further multivariate logistic analyses were carried out in different cohorts. Results are shown in Table 2. The final logistic model included 8 independent predictors Age, Alb, Digestive tract reconstruction, Stenosis site, Diabetes, ERCP operation time (h), obstruction length and Cholangioscopy was developed as a simple-to-use nomogram, which is illustrated in Figure 4.

**Table 2:** Results of multivariate logistic regression for training cohort.

Characteristic	*N*	Event *N*	OR	95% CI	*p*-value
Age (years)					
<60	427	70	—	—	
≥60	342	81	1.64	1.10, 2.43	0.014
Alb (g/L)					
<35	443	112	—	—	
≥35	326	39	0.56	0.35, 0.88	0.014
Etiologie					
Ampullary carcinoma	47	5	—	—	
Autoimmune	22	7	3.09	0.74, 13.48	0.122
Benign strictures	223	22	0.64	0.22, 2.13	0.426
d-CCA	65	11	1.50	0.46, 5.47	0.512
GBC	39	10	1.49	0.40, 6.04	0.562
h-CCA	122	38	1.47	0.42, 5.83	0.561
HCC	43	14	1.37	0.34, 6.03	0.667
Iatrogenic	22	5	1.06	0.23, 4.90	0.938
Metastatic tumors	44	12	0.98	0.25, 4.20	0.979
Pancreatic cancer	142	27	1.41	0.50, 4.69	0.544
Digestive tract reconstruction					
No	738	135	—	—	
Yes	31	16	5.21	2.16, 12.82	<0.001
Stenosis site					
Extrahepatic	498	73	—	—	
Hilar/Intrahepatic	271	78	1.75	0.85, 3.57	0.127
Diabetes					
No	626	105	—	—	
Yes	143	46	2.48	1.57, 3.89	<0.001
Obstruction length (cm)					
>2.5	337	85	—	—	
≤2.5	432	66	0.69	0.46, 1.02	0.065
Cholangioscopy					
No	534	77	—	—	
Yes	235	74	2.63	1.76, 3.92	<0.001

CI, Confidence Interval; OR, Odds Ratio.

### Validation of the model

**Figure 5:**
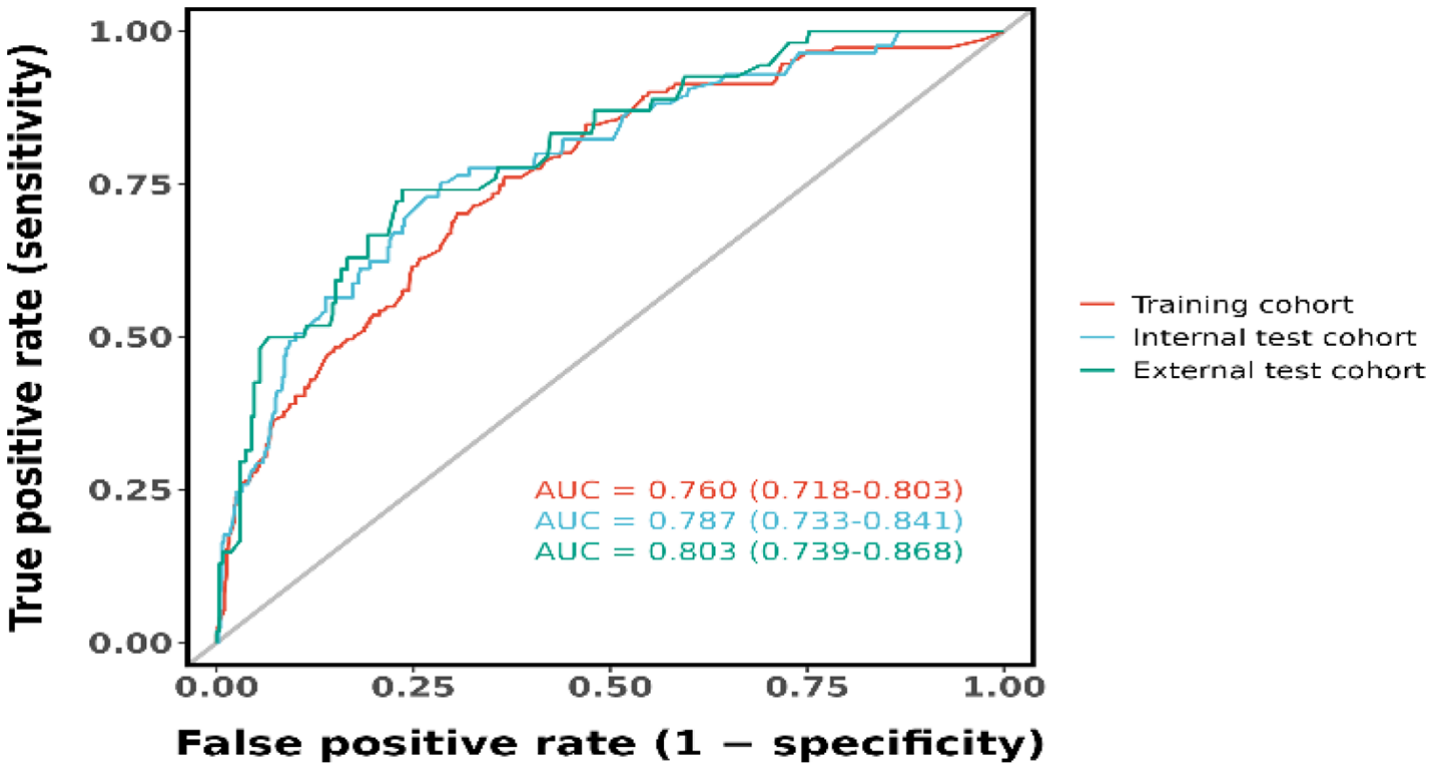
ROC curves of the nomogram prediction model.

**Figure 6:**
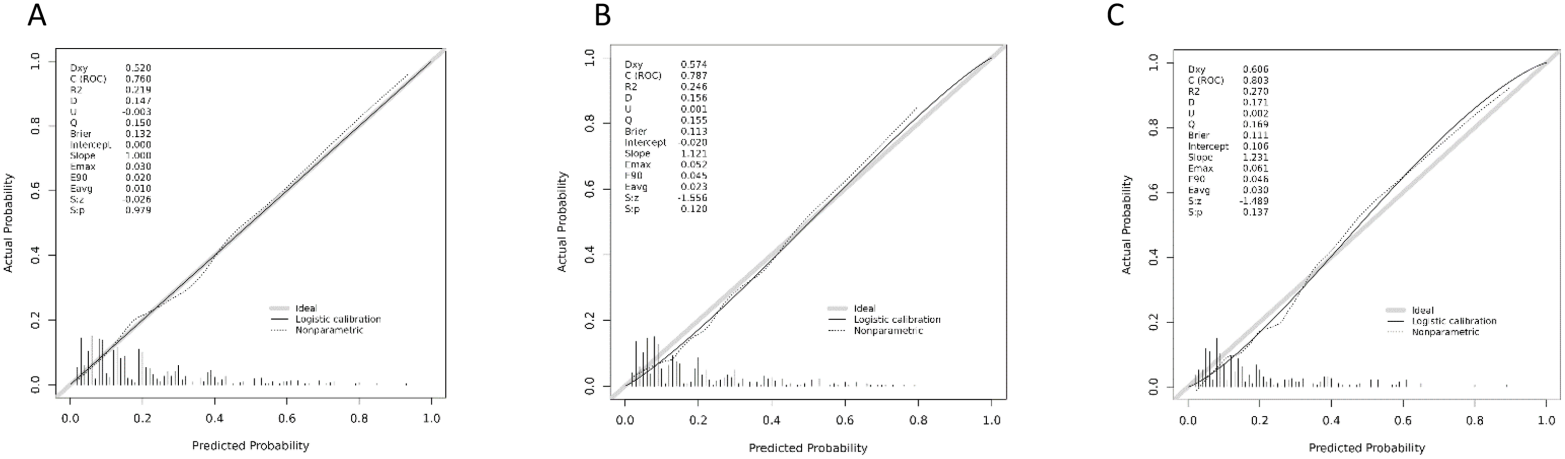
**(A)** Calibration curve based on the training set. **(B)** Calibration curve based on the internal validation set. **(C)** Calibration curve based on an external validation set.

The figures below display the AUCs of the model in different cohorts. The pooled area under the ROC curve of the nomogram for the predictive model was 0.760 in the training set and 0.787 in the validation set, indicating strong performance (Figure 5). Calibration plots of the nomogram in various cohorts show a positive correlation between observed and predicted cholangitis (Figure 6). These results confirm that the original nomogram remains valid for use in validation sets, with its calibration curve closely resembling the ideal curve and demonstrating consistent predicted results with actual findings.

### Decision curve analysis

**Figure 7:**
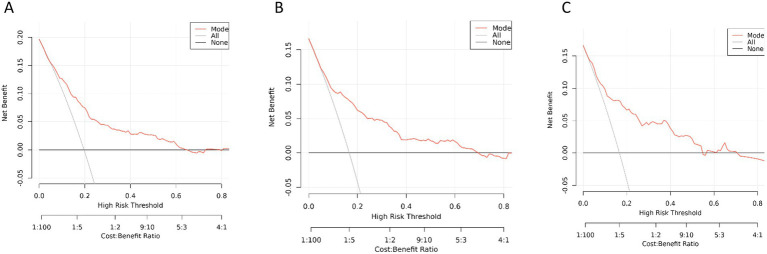
Decision curve analysis was used to determine the clinical utility of column line plots for predicting PEC. Black line = net benefit when all patients are considered as not having PEC. Grey line = net benefit when all patients are considered as having PEC. The red curve represents the net benefit of the nomogram. **(A)** In the training cohort. **(B)** In the internal test cohort. **(C)** In the external test cohort.

Figure 7 displays the DCA curves related to the nomogram. This research shows that the nomogram offers substantial net benefits for clinical application through its DCA curve. The Decision Curve Analysis (DCA) demonstrated that our model provides a positive net benefit across a wide range of threshold probabilities. Considering the high baseline risk of PEC in biliary stricture patients and the severity of potential outcomes, a threshold probability range of 10 to 40% is deemed clinically reasonable for initiating prophylactic antibiotics. Within this range, our model outperformed both the “treat-all” and “treat-none” strategies, offering a superior balance between sensitivity and specificity.

## Discussion

Biliary stricture can be divided into benign stenosis and malignant stenosis. The causes of benign stenosis include bile duct inflammation, bile duct stones, and iatrogenic bile duct injury, etc. The causes of malignant stenosis include liver cancer, bile duct cancer, pancreatic cancer, and so on (2, 12, 13). Patients with biliary strictures are more likely to develop cholangitis (14, 15). Patients with biliary stricture often require ERCP procedures for diagnosis and treatment (3, 16, 17). ERCP is an invasive operation, and due to the complex structure of duodenoscopes, leads to more complications, such as PEC (18, 19). The occurring incidence ranges from 1% to 5% (20–22). PEC incidence can reach up to 20% in patients with biliary stricture (23). PEC can delay the length of hospital stay, increase the economic burden of patients, and even threaten the life of patients. If we can assess the risk of PEC in patients with biliary stricture early, we can treat it as early as possible and avoid the progression of the disease.

In the current study, we developed and validated a nomogram for predicting PEC, based on a cohort of biliary stricture patients. The main predictors incorporated into the nomogram included Age, Alb, digestive tract reconstruction, etiology, stenosis site, diabetes, obstruction length, ERCP operation time and the use of cholangioscopy, which were statistically significant in multivariate logistic regression analysis. To our knowledge, this is the first study to predict the risk of cholangitis after ERCP in patients with biliary strictures.

ESGE suggested that hilar obstruction, primary sclerosing cholangitis, and cholangioscopy were the high-risk factors for PEC (24). In a recent retrospective study, age over 60 years and the history of ERCP is an independent risk factor for PEC (25). Whereas previous studies have focused on non-selective populations, our study was performed on patients diagnosed with specific biliary strictures.

Our study was more specific and focused on patients with biliary stricture to evaluate the situation of cholangitis after ERCP. Variables examined in the study includes the basic characteristics of the patients, the characteristics of stenosis, and ERCP-related operations. We found that Age, Alb, digestive tract reconstruction, stenosis site, diabetes, obstruction length, ERCP operation time (h), and the use of cholangioscopy were the risk factors for PEC in biliary strictures. We combined these variables to establish and validate a novel predictive tool for PEC for patients with biliary strictures.

The nomogram of this study provides several clinical significances. First, it provides clinicians with a quantitative tool that can predict the risk of PEC more accurately than traditional methods, contributing to better risk stratification. In addition, early identification of high-risk individuals by this nomogram could lead to timely interventions and potentially reduce morbidity and mortality. There are some risk factors that we cannot change. For example, the Location of Stricture and the Length of the Stricture cannot be changed. Some factors, such as good control of the blood glucose and elevated level of Alb can reduce the occurrence of PEC.

Who should receive antibiotic prophylaxis before ERCP remains controversial (26–28). Some believed that prophylactic antibiotics are not necessary for all patients before ERCP and may have little effect on the prevention of cholangitis, but may reduce the risk of bacteremia (27, 29). The ESGE guidelines state that preoperative prophylactic antibiotics can be used in patients with expected inadequate biliary drainage, and severe immune insufficiency, and when choledochoscopy is performed (24). We suggest that the PEC high-risk patients with bile duct stricture undergo preoperative use of antibiotics to prevent infection. The clinically relevant threshold for initiating prophylaxis in this group is shifted upwards (estimated 5%–25%). Our DCA demonstrates that even within this high-risk context, a “treat-all” strategy is not optimal, particularly when the acceptable risk threshold exceeds 18%. Our model provides superior net benefit in this range, supporting a precision medicine approach to antibiotic prophylaxis even among high-risk subgroups.

Patients with hilar biliary strictures as well as intrahepatic strictures have a higher risk of developing PEC than patients with extrahepatic strictures. PSC, ICC, h-CCA, and metastatic tumors are the main causes of hilar strictures and intrahepatic strictures. These patients have long, multi-vessel biliary strictures and are in poor physical condition, requiring multiple stents for drainage.

Although the use of choledochoscopy may increase the risk of PEC, choledochoscopy is of great value in the differential diagnosis of benign and malignant biliary strictures (30). SpyGlass-guided visual impression combined with SpyBite was better for differential diagnosis of benign and malignant bile duct strictures in terms of sensitivity and accuracy compared with conventional endoscopic diagnostic methods such as cytobrush (31, 32). Furthermore, Spyglass can assist the operator in performing selective cannulaiton in cases where the bile duct is severely narrowed (33–36). Literature and guidelines mention the use of antibiotics in patients undergoing choledochoscopy, especially elderly patients and those with prior stent placement or intraduct lithotripsy (37). Othman et al. (38) reported that the use of choledochoscopy may be associated with an 8.8% incidence of bacteremia and a 7% incidence of cholangitis. In our study, it was found that the probability of PEC caused by choledochoscopy in patients with biliary stricture was even more than 20%. This might be due to the fact that in our research cohort, there were a greater number of cases of hilar bile duct stenosis, and the proportion of difficult ERCP procedures was also higher. Using antibiotics before the surgery and reducing the amount of fluid injected during the operation may be effective methods to reduce the infection rate caused by choledochoscopy (39). However, more research data is needed to support the viewpoint.

Although our study does not suggest the predictive value of EST and EPBD, it does not mean that these procedures have no effect. In our study, minor EST (3–5 mm)with or without small EPBD (6–8 mm) was used to avoid the destruction of the papillary sphincter and reduce the risk of retrograde infection.

In addition, we found that digestive tract reconstruction increases the risk of PEC. Common digestive tract reconstruction surgery includes subtotal gastrectomy, bilioenteric anastomosis, pancreaticoduodenectomy, and so on (40). These operations not only increase the difficulty of endoscopic intervention but also are associated with a high incidence of postoperative complications. Some patients may require Endoscopic Ultrasound-Guided Biliary Drainage (EUS-BD) due to the failure of conventional ERCP. However, the relationship between EUS-BD and PEC warrants further investigation.

Although our study has achieved some success, it also has some limitations. In this model, operator experience may vary across hospitals. If the operator is inexperienced, this may further increase the risk of PEC in patients with biliary strictures.

Future research should aim to externally validate our nomogram in diverse populations and settings. Additionally, integrating novel predictors or biomarkers can enhance the predictive accuracy of the nomogram, which deserves further investigation.

## Limitations

Our Decision Curve Analysis focused specifically on the prevention of Post-ERCP Cholangitis (PEC) as the primary outcome. While this reflects the critical importance of preventing sepsis in patients with biliary strictures, we did not explicitly incorporate competing harms from other potential complications (e.g., post-ERCP pancreatitis, pneumonia) or specific antibiotic-related adverse events into the net benefit calculation. Consequently, the estimated net benefit primarily applies to clinical scenarios where preventing PEC is the dominant decision driver. Future studies incorporating a multi-outcome framework could provide a more holistic assessment of clinical utility.

## Data Availability

The datasets presented in this article are not readily available because they contain detailed clinical data of hospitalized patients, and sharing these data publicly would compromise patient privacy. Requests to access the datasets should be directed to the corresponding author, Jiangfeng Hu (doctorhjf@foxmail.com).
