# Supplementary figures and images for: Clinical predictive modeling for post-ERCP cholangitis in biliary stricture patients
Source: Front Med (Lausanne). 2026 Apr 22;13:1738706. doi: 10.3389/fmed.2026.1738706 (PMC13143609; doi:10.3389/fmed.2026.1738706)

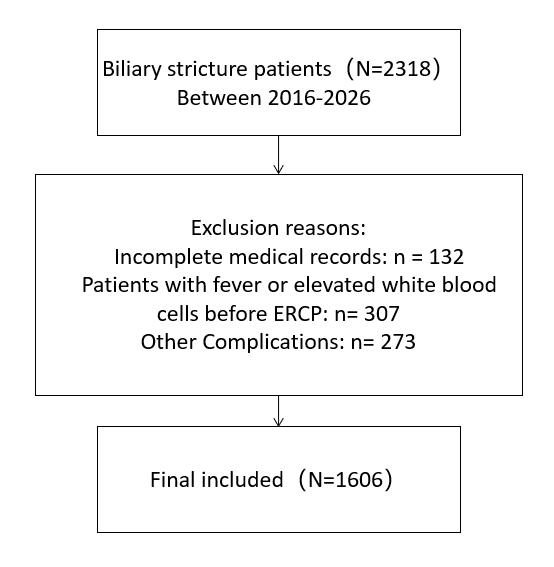

Supplement: Supplementary file 1 [file Image_1.jpg]
